# Pediatric Palatal Fibroma

**DOI:** 10.5005/jp-journals-10005-1414

**Published:** 2017-02-27

**Authors:** Rahul Mishra, Tayyeb S Khan, Tarannum Ajaz, Mamta Agarwal

**Affiliations:** 1 Assistant Professor, Department of Dentistry, University of Medical Sciences, Saifai Etawah, Uttar Pradesh, India; 2 Reader, Department of Oral and Maxillofacial Surgery, Purvanchal Institute of Dental Sciences, Gorakhpur, Uttar Pradesh, India; 3 Reader, Department of Prosthodontics, Purvanchal Institute of Dental Sciences, Gorakhpur, Uttar Pradesh, India; 4 Professor, Department of Oral and Maxillofacial Surgery, Purvanchal Institute of Dental Sciences, Gorakhpur, Uttar Pradesh, India

**Keywords:** Oral habits, Palatal fibroma, Surgical pedodontics.

## Abstract

Fibroma is one of the most common soft tissue benign tumors of the oral cavity. These masses represent hyperplasias instead of true neoplasm, which develop due to irritation to the mucosal tissue resulting in proliferation of the cells. Although so common in the oral cavity, its occurrence on the palate is rare, mainly due to fewer chances of trauma. Here, we report a case of palatal fibroma in a child diagnosed on the basis of clinical, radiological, and histological features. The case represents an extremely rare occurrence as unusual trauma due to thumb sucking seemed to be the only apparent traumatic factor in the palatal region.

**How to cite this article:**

Mishra R, Khan TS, Ajaz T, Agarwal M. Pediatric Palatal Fibroma. Int J Clin Pediatr Dent 2017; 10(1):96-98.

## INTRODUCTION

Fibroma or focal fibrous hyperplasia of the oral mucosa is the most common benign neoplasm of the oral cavity. According to Torres-Domingo et al,^1^ out of 300 benign tumors of the oral mucosa, 53% were histologically diagnosed as fibroma, and it is the most frequently found benign tumor of the oral cavity.

Fibromas are hyperplasias of fibrous connective tissue in response to local irritation or trauma. Tissue enlargements attributable to injury represent a hyper-plastic reaction and are collectively grouped as “reactive proliferations.” It is also known as irritational fibroma, traumatic fibroma, fibrous nodule, or fibroepithelial polyp.^2^ It was first reported in 1846 as fibrous polyp and polypus and is found in 1.2% of adults.^3,4^

The diagnosis of these lesions is based mainly on his-topathological features. Most of these lesions are relatively characteristic in presentation, leaving very little doubt about the diagnosis. In certain instances, however, unusual findings may result in diagnostic uncertainty. Here, we report a rare case of palatal fibroma occurring in an 8-year-old boy where the causative irritational factor was due to a seemingly innocuous parafunctional habit of thumb sucking.

## CASE REPORT

An 8-year-old boy reported to the Department of Pedodontics and Preventive Dentistry at our center with a chief complaint of palatal growth in the midline region. History revealed that the problem started with ulceration in the palate about 3 years back. After a few days, he observed a small growth in the same region, which gradually enlarged during the following months. Patient also reported of thumb sucking habit till the age of 5 years. He was advised antibiotic and antifungal medication by the local physician to which there was no response.

**Fig. 1:**
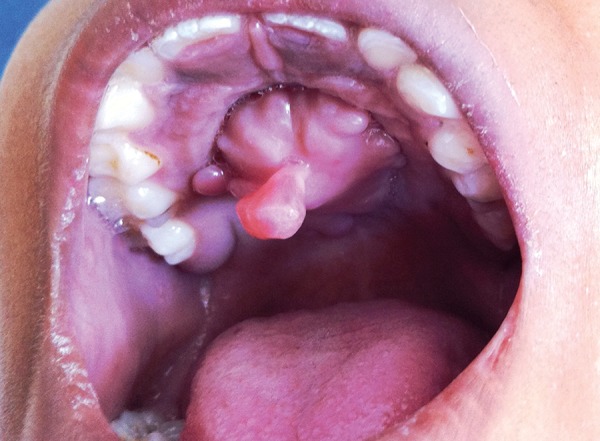
Preoperative view with palatal fibroma

On intraoral examination, a large, smooth-surfaced, and pedunculated growth was observed (2.5 × 2 × 1.5 cm approx) in the palatal area (Fig. 1). On palpation, the outgrowth was soft, nontender, and attached with a stalk to the palatal mucosa. Ipsilateral submandibular lymph nodes were enlarged, palpable, and nontender.

### Investigation

- Radiographically, no abnormality was seen.
- Routine blood investigations were within normal range.

**Fig. 2:**
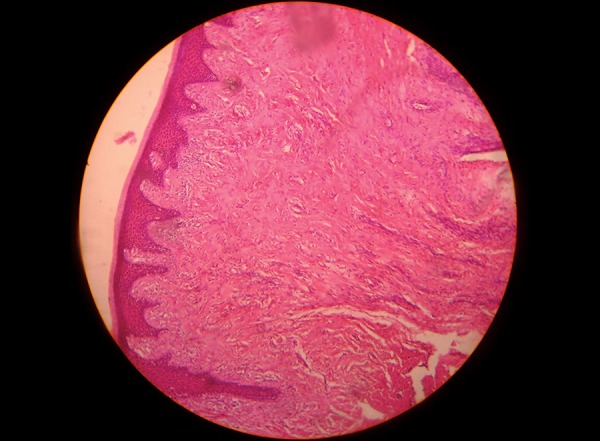
Histopathological view

Incisional biopsy was done, and the tissue was sent for histopathalogic examination. The report showed orthokeratinized stratified squamous epithelium with short rete pegs encircling the connective tissue stroma with abundant collagen fiber bundles along with proliferating spindle-shaped fibroblasts, few myxomatous areas, chronic inflammatory cells infiltration, and blood vessels (Fig. 2). Overall, the histopathological features were suggestive of fibroma.

### Differential Diagnosis

Clinically, the soft tissue overgrowth appeared of normal mucosal color and texture, and due to the specific location, the possible differential diagnosis included salivary gland tumors, giant cell fibroma, myxoma, pyogenic granuloma, and neurofibroma. As the site of soft tissue growth was at the midline of posterior hard palate, the possibility of irritation fibroma was not considered prior to the histopathological report.

**Figs 3A and B:**
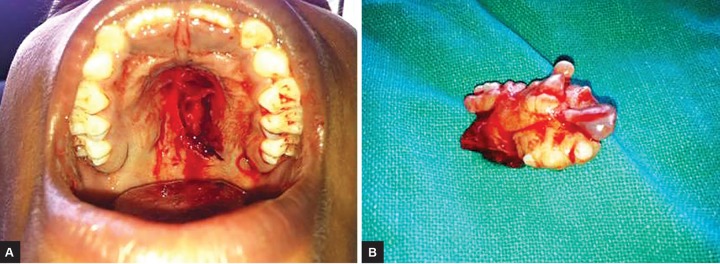
Intraoperative view and excised lesion

**Figs 4A and B:**
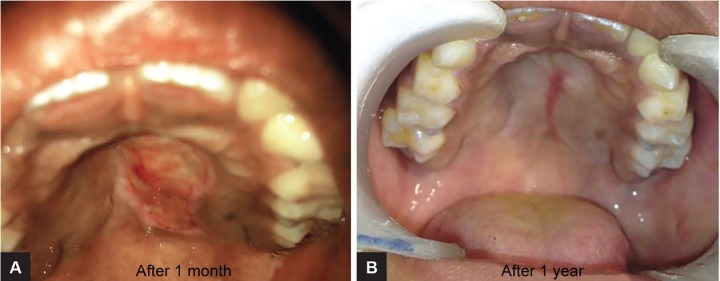
Postoperative views after 1 month and 1 year

Final diagnosis of irritational fibroma was made and surgical excision of lesion was done (Figs 3A and B). Follow-up showed perfect healing and no recurrence until 1 year postsurgery (Figs 4A and B).

## DISCUSSION

Irritational fibroma is usually sessile, round or ovoid, nontender and may be lighter in color than the surrounding tissue due to reduced vascularity. Due to the gradual and slow growth of the lesion, the patients are generally aware of the mass.

The irritational fibroma has a 66% female predilection and can occur at any age, but is usually seen in the 4th to sixth decades of life.^5^ Contrary to the aforementioned evidence, this case presents an 8-year-old male child with the lesion. The presumed etiology of fibroma is trauma to the affected mucosa. In the case presented, although there was no direct history of trauma, a traumatic stimulus could have been inflicted due to parafunctional habit of thumb sucking or maybe due to trauma from any sharp foreign object.^6^ The patient did report of initial ulceration prior to the growth formation. Although the irritational fibroma can occur anywhere in the oral cavity, the most common site is the buccal mucosa along the occlusal line; other common sites are labial mucosa, tongue, and gingiva. Midline palatal fibroma at such a young age has not been reported till date in literature, except in old denture wearers, where it presents as a flat, pancake-shaped mass.

As far as size is concerned, the lesions generally are less than 1 cm in diameter.^7,8^ In contrast to the common clinical presentation, the present lesion was of a much larger dimension than normal, covering about two-thirds of the hard palate.

The pathological mechanism of fibroma involves hyperplasia due to trauma to the mucosal tissue resulting in proliferation of cells followed by collagen fibrillogen-esis. In the oral cavity, apart from fibroblasts, the periodontal tissues, fibrovascular connective tissues, periosteum, etc. may be the target of injury.^9^ Pyogenic granulomas arise from proliferation of the fibrovascular connective tissue, whereas peripheral giant cell granulomas arise from proliferation of the periosteal tissue containing osteoblasts. Periodontal ligament fibroblast proliferation gives rise to peripheral ossifying fibroma as they retain the potential to form bone and cementum.^9^ In our case, the absence of periodontal tissue ruled out the possibility of peripheral ossifying fibroma as a possible diagnosis.

Microscopically, nodular deposition of dense collagen in association with chronic inflammation, spindle-shaped fibroblast, and overlying thinning mucosa is present, which confirmed the diagnosis. Trauma-related changes, such as hyperkeratosis and ulceration may also be seen in long-standing fibromas, which were not present in our case.^7,8^ Fibroma does not have any malignant potential, and recurrence is rare following total excision.

Fibromas, though very common lesions of the oral cavity and characteristic in presentation, may sometimes pose a diagnostic challenge. Clinicians should consider the possibility of diagnosing irritation fibroma in younger age groups and in unusual locations as palate. Detailed history regarding the lesion, precise clinical workup combined with microscopic presentation is required for diagnostic confirmation and proper management of such cases.

## CONCLUSION

Fibrous growths of the oral soft tissues are fairly common and include a diverse group of reactive and hyperplastic conditions. As a pedodontic, the key for management should be early education and interception of abnormal oral habits in children followed by identification of any reactive hyperplastic lesion by devising a differential diagnosis to enable precise patient evaluation and, thereon, its treatment.

## References

[1] Torres-Domingo S, Bagan JV, Jiménez Y, Poveda R, Murillo J, Díaz JM, Sanchis JM, Gavaldá C, Carbonell E (2008). Benign tumors of the oral mucosa: a study of 300 patients.. Med Oral Patol Oral Cir Bucal.

[2] Toida M, Murakami T, Kato K, Kusunoki Y, Yasuda S, Fujitsuka H (2001). Irritational fibroma of the oral mucosa: a clini-copathological study of 129 lesions in 124 cases.. Oral Med Pathol.

[3] Tomes J (1846). A course of lectures on dental physiology and surgery (lectures I-XV).. Am J Dent Sci.

[4] Bouquot JE, Gundlach KK (1986). Oral exophytic lesions in 23,616 white Americans over 35 years of age.. Oral Surg Oral Med Oral Pathol.

[5] Barker DS, Lucas RB (1967). Localised fibrous overgrowths of the oral mucosa.. Br J Oral Surg.

[6] Singh S, Subba Reddy VV, Dhananjaya G, Patil R (2004). Reactive fibrous hyperplasia associated with a natal tooth.. J Indian Soc Pedod Prev Dent.

[7] Shafer WG., Hine MK., Levy BM. (2009). A textbook of oral pathology..

[8] Neville BW., Damm DD, Allen CM., Bouquot J. (2009). Oral and maxillofacial pathology..

[9] Silverman S., Eversole LR., Truelove EL. (2001). Essentials of oral medicine..

